# A Four-Gene Panel in Rectal Swab Samples as a Biomarker for Colorectal Cancer Screening

**DOI:** 10.3390/cells13110930

**Published:** 2024-05-28

**Authors:** Lui Ng, Sunny Kit-Man Wong, Hung-Sing Li, Ryan Wai-Yan Sin, Johnny Hon-Wai Man, Oswens Siu-Hung Lo, Roberta Wen-Chi Pang, Dominic Chi-Chung Foo, Wai-Lun Law

**Affiliations:** Department of Surgery, School of Clinical Medicine, Li Ka Shing Faculty of Medicine, The University of Hong Kong, Hong Kong SAR, China; h0994148@hku.hk (S.K.-M.W.); lhsing2@hku.hk (H.-S.L.); ryansin@connect.hku.hk (R.W.-Y.S.); johnnyb@hku.hk (J.H.-W.M.); oswenslo@yahoo.com (O.S.-H.L.); robertap@hku.hk (R.W.-C.P.); ccfoo@hku.hk (D.C.-C.F.)

**Keywords:** colorectal cancer, biomarker, rectal swab, polyps

## Abstract

Background: The dysregulation of gene expression is one of the key molecular features of colorectal cancer (CRC) development. This study aimed to investigate whether such dysregulation is reflected in rectal swab specimens of CRC patients and to evaluate its potential as a non-invasive approach for screening. Methods: We compared the expression level of 14 CRC-associated genes in tumor and adjacent non-tumor tissue of CRC patients and examined the correlation of their levels in tissue with paired rectal swab specimens. The level of these 14 genes in rectal swab specimens was compared among patients with CRC or polyp and control subjects, and the diagnostic potential of each dysregulated gene and the gene panel were evaluated. Results: The expression of *CXCR2*, *SAA*, *COX1*, *PPARδ*, *PPARγ*, *Groγ*, *IL8*, *p21*, *c-myc*, *CD44* and *CSF1* was significantly higher in CRC, and there was a significant correlation in the levels of most of them between the CRC and rectal swab specimens. In the training study, we showed that *CD44*, *IL8*, *CXCR2* and *c-myc* levels were significantly higher in the rectal swab specimens of the CRC patients. Such result was confirmed in the validation study. A panel of these four genes was developed, and ROC analysis showed that this four-gene panel could identify CRC patients with an AUC value of 0.83 and identify overall polyp and precancerous adenoma patients with AUC values of 0.6522 and 0.7322, respectively. Finally, the predictive study showed that the four-gene panel demonstrated sensitivities of 63.6%, 76.9% and 88.9% in identifying overall polyp, precancerous adenoma and CRC patients, respectively, whereas the specificity for normal subjects was 72.2%. Conclusion: The expression of CRC-associated genes in rectal swab specimens reflects the dysregulation status in colorectal tissue, and the four-gene panel is a potential non-invasive biomarker for early precancerous adenoma and CRC screening.

## 1. Introduction

Colorectal cancer (CRC) is one of the major healthcare problems worldwide. It is the third most common cancer in males and the second most common in females, with an estimated incidence of 1.4 million new cases and a mortality of 693,900 deaths in 2012 [1]. The incidence and death rates of CRC are rising quickly in Asian countries [2]. Detecting CRC in its early stages is crucial for successful treatment, but most patients do not show any symptoms at this stage. Only 39% of CRC patients are diagnosed at the localized stage, while 56% are diagnosed at the regional or distant stage [3]. Therefore, early screening for polyps and CRC is vital in reducing its occurrence and mortality rate [4].

Colonoscopy is considered the most reliable method for diagnosing colon cancer, but many individuals are hesitant to undergo this procedure due to reasons such as embarrassment, preparation, fear of pain and discomfort. Additionally, the cost, invasiveness, potential complications and requirement for expertise make it impractical for mass screening [5]. Therefore, there is a need for a non-invasive test that can accurately identify individuals at risk of having colon cancer, thus sparing those without polyps from undergoing unnecessary procedures. The fecal occult blood test (FOBT) is a relatively simple and affordable test, although its sensitivity and specificity are not ideal. However, the results from several large-scale studies conducted over up to 30 years consistently demonstrate that implementing FOBT screening can reduce CRC mortality by 12% to 33% [5,6,7,8]. These findings indicate that a screening tool with higher sensitivity and specificity could further decrease the mortality rate.

Extensive research has been conducted on molecular biomarkers in CRC. CRC is a complex disease that occurs due to epigenetic and genetic changes in various genes involved in oncogenesis, tumor suppression, mismatch repair and cell cycle regulation in the cells of the colon mucosa [9]. These alterations collectively contribute to the initiation and progression of CRC through a multistep process known as the adenoma–carcinoma sequence. Studies have demonstrated that biopsy samples taken from the apparently normal colon mucosa of individuals with polyps or a personal or family history of cancer exhibit different gene expression patterns compared to those of controls without polyps or cancer history [10,11]. Additionally, gene expression profiles obtained from rectal swab samples closely resemble those obtained from biopsies [12]. These findings suggest that the gene expression profile in rectal swab specimens may serve as a non-invasive biomarker for screening CRC patients.

The objective of this study is to examine whether the abnormal gene expression patterns observed in CRC can be detected in rectal swab specimens from CRC patients. Additionally, we aim to assess the potential of utilizing the gene expression profile obtained from rectal swabs as a non-invasive biomarker for diagnosing CRC.

## 2. Materials and Methods

### 2.1. Patient Tissue and Rectal Swab Specimens

The Institutional Review Board (IRB) of the University of Hong Kong has granted approval for the human sample collection protocol, and all clinical investigations have been carried out in compliance with the principles outlined in the Declaration of Helsinki. Informed written consent has been obtained from all participants. Tissue samples were collected from 21 patients, promptly frozen in liquid nitrogen and stored at −80 °C until analysis. The patient database of our hospital was utilized to gather clinicopathological data. The rectal swab study consisted of 307 Chinese patient subjects who underwent colonoscopies between 2011 and 2014 at Queen Mary Hospital, Hong Kong. The training study consisted of 39 normal subjects and 37 CRC patients. The validation study consisted of 60 normal subjects, 68 polyp patients and 45 CRC patients. The blinded study consisted of 58 subjects of unknown clinical data prior to measuring the gene level. The age and gender information for the subjects recruited is shown in Table 1, and the diagnosis and stage information for CRC patients is shown in Table 2. Rectal swab specimens were collected from patients prior to their colonoscopy examination. Colon mucosal cells were collected from individuals using an anoscope, which involved inserting a soft brush approximately 2 cm into the colon and gently swabbing the area. The collected cells were then extracted from the brush by dipping and swirling it in RNase later buffer.

### 2.2. RNA Extraction and Preparation of RNA

Tissues were subjected to total RNA extraction using the PurelinkTM RNA Mini kit (Ambion, Austin, TX, USA), following the instructions provided by the manufacturer. To eliminate any potential genomic DNA contamination, the RNA samples were treated with RNase-free DNase. The quantity and quality of the extracted RNA were assessed using the NanoDrop 2000 (Thermo Scientific, Waltham, MA, USA).

### 2.3. cDNA Synthesis and Quantitative Real-Time Polymerase Chain Reaction

For reverse transcription, 1 μg of tissue RNA or 50 ng of rectal swab RNA was utilized, and the PrimeScript first-strand cDNA Synthesis Kit (Takara, Tokyo, Japan) was employed in accordance with the manufacturer’s instructions. Subsequently, quantitative PCR amplification was performed using specific oligonucleotide primers and the SYBR^®^ Premix Ex Taq™ reagent with ROX plus (Takara, Tokyo, Japan), following the manufacturer’s protocol. Fourteen genes that were involved in the pathogenic pathway of CRC were chosen, including *CXCR2*, *SAA*, *COX1* and COX2, PPAR-α, -δ and - γ, Gro-α and -γ, *IL8*, *p21*, *c-myc*, *CD44* and *CSF1*. Real-time PCR was carried out using the ABI 7900HT Fast Real-Time PCR System (Applied Biosystems, Foster, CA, USA) at 95 °C for 30 s, followed by 40 cycles at 95 °C for 3 s and at 60 °C for 30 s. The gene level of the actin was used for normalization. Each assay was carried out in triplicate.

### 2.4. Statistical Analysis

To compare the differences in target mRNA expression between the tumor and adjacent non-tumor tissue of CRC patients, a paired *t*-test was employed. Pearson correlation analysis was used to examine the correlation between paired tissue and rectal swab specimens of CRC patients. The Mann–Whitney test was utilized to compare the differences in target mRNA expression among rectal swab specimens of CRC patients, polyp patients and normal subjects. Receiver operating characteristic (ROC) curves and the area under the ROC curve (AUC) were employed to assess the sensitivity and specificity of gene biomarkers, as well as to determine the cut-off value for the diagnosis of CRC. Logistic regression was employed to develop a combined mRNA panel for the diagnosis of CRC. All statistical analyses were performed using SigmaPlot version 10.0 (Systat Software Inc., San Jose, CA, USA), with a significance level set at *p* ≤ 0.05.

## 3. Results

### 3.1. Correlation of 14 Genes’ Expression between CRC and Rectal Swab Specimens

**Figure 1:**
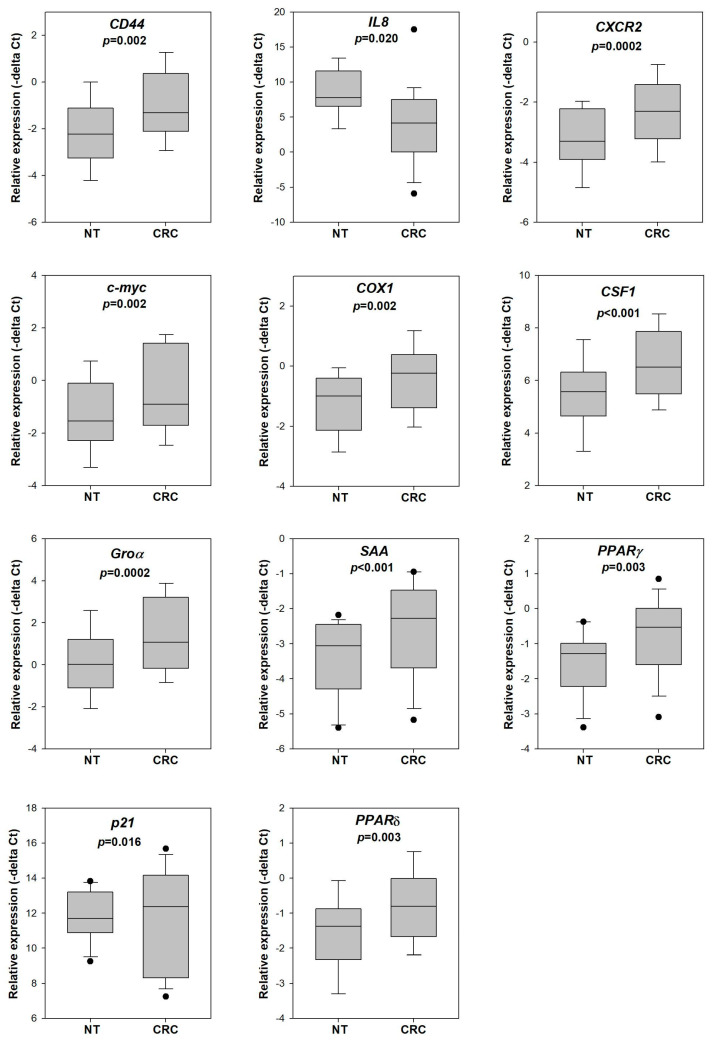
Gene expression in tumor and adjacent non-tumor tissue of CRC patients. The expression of *CXCR2*, *SAA*, *COX1*, *PPARδ*, *PPARγ*, *Groγ*, *IL8*, *p21*, *c-myc*, *CD44* and *CSF1* was significantly higher in the tumor tissue (CRC) when compared to the adjacent non-tumor tissue (NT) of CRC patients (n = 21). The relative expression of each gene was calculated by delta Ct (target gene-actin), and the *p*-value was determined by a paired *t*-test.

We applied quantitative PCR to determine the expression of 14 genes including *CXCR2*, *SAA*, *COX1* and COX2, PPAR-α, -δ and -γ, Gro-α and -γ, *IL8*, *p21*, c-*myc*, *CD44* and *CSF1* in tumor and adjacent normal mucosa of 21 CRC patients. As shown in Figure 1, except for PPAR-α, Gro-α and COX2, all the genes were significantly overexpressed in CRC when compared to the paired non-tumor normal mucosa. PPAR-α and Gro-α also showed a trend of overexpression in CRC, with *p*-values slightly above 0.05 (*p* = 0.075 and 0.068, respectively). In line with previous reports, these results showed that the genes above were dysregulated in CRC.

**Figure 2:**
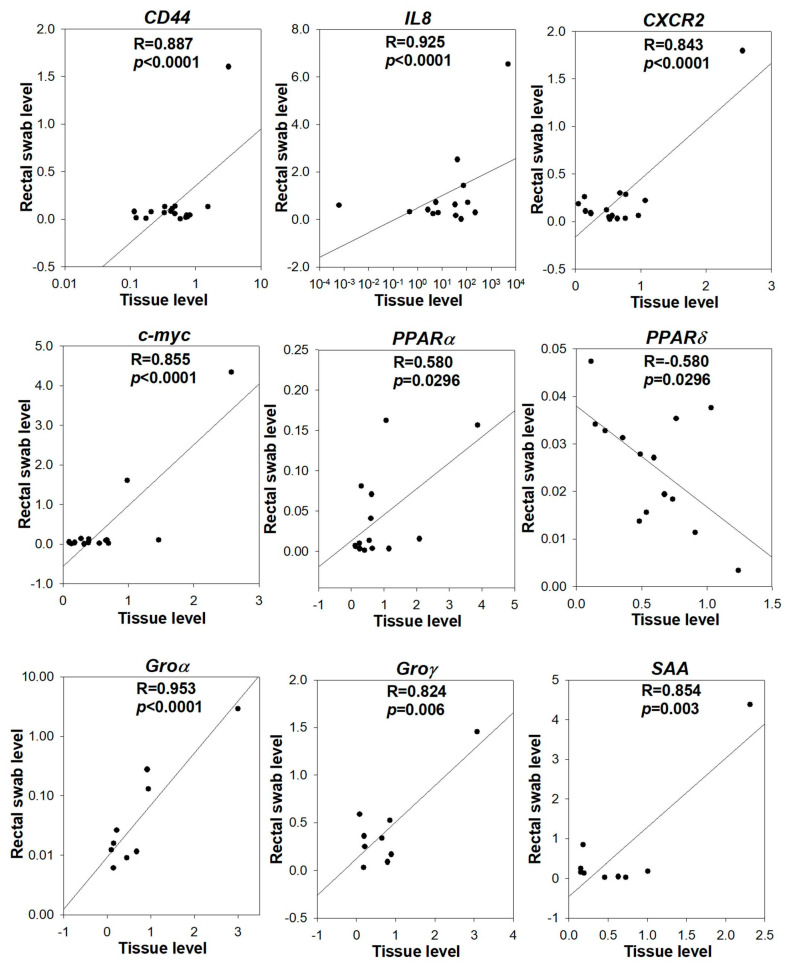
Correlation of gene expressions between rectal swab and tissue specimens. The gene expression of *CD44*, *IL8*, *CXCR2*, *c-myc*, PPARα, *PPARδ*, Groα, *Groγ* and *SAA* in the tissue was significantly correlated with that in the paired rectal swab of CRC patients (N = 21). The gene level in the tissue was expressed as a fold change of its level in tumor to non-tumor, whereas the gene level in the rectal swab was expressed as a fold to actin. The correlation coefficient (R) and *p*-value were analyzed by Pearson correlation.

We detected the expression of the 14 genes in paired rectal swab specimens from the above 21 CRC patients and examined if the gene expression in rectal swab specimens was correlated with that in tissue specimens. Our results demonstrated that 9 genes out of the 14 genes tested, including *CD44*, *IL8*, *CXCR2*, *c-myc*, PPARα, *PPARδ*, Groα, *Groγ* and *SAA*, showed a significant correlation between rectal swab and tissue specimens (Figure 2), despite *PPARδ* among them showing an inverse correlation. Nevertheless, these results suggested that the dysregulation of the gene in CRC was reflected in the rectal swab specimens; hence, the potential of their level in rectal swab specimens as a diagnostic biomarker warranted further investigation.

### 3.2. Expression of the 14 Genes in the Rectal Swab Specimens of Normal Subjects and CRC Patients of the Training Set

**Figure 3:**
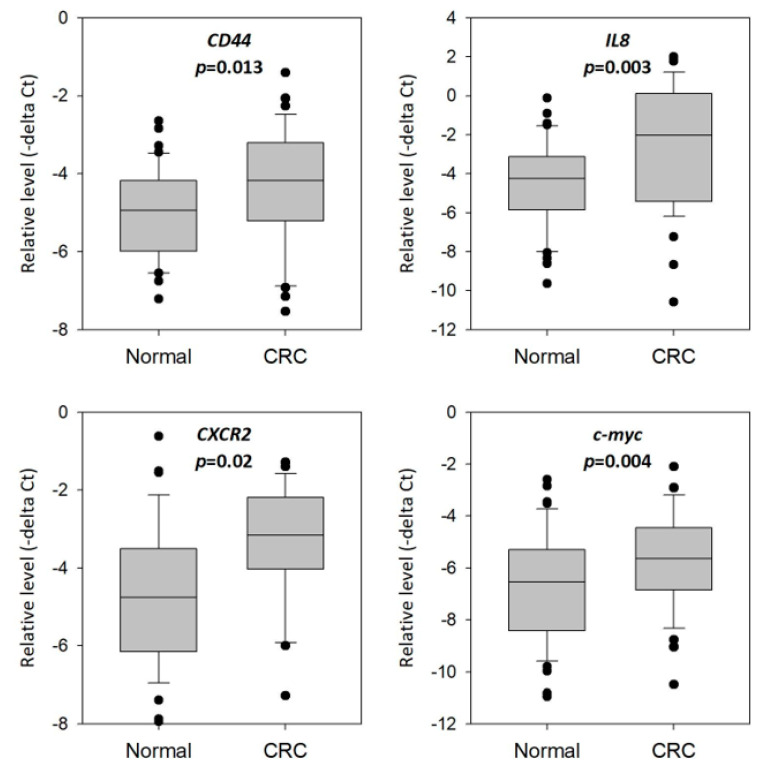
Significantly higher levels of *CD44*, *IL8*, *CXCR2* and *c-myc* in the rectal swab specimens of CRC patients when compared to healthy controls in the training study. The expression of *CD44*, *IL8*, *CXCR2* and PPARα in the rectal swab specimens of CRC (*n* = 37) and control subjects (*n* = 39) was compared in the training study. *p*-values were calculated using the Mann–Whitney test.

We compared the expression of the 14 genes in rectal swab specimens between normal and CRC patients in the training set consisting of 39 normal subjects and 37 CRC patients. *CD44*, *IL8*, *CXCR2* and *c-myc* were significantly overexpressed in CRC patients when compared with normal subjects (Figure 3). The fold changes of these genes in CRC patients compared to those of normal subjects were as follows: *CD44* (1.84-fold; *p* = 0.013); *IL8* (3.69-fold; *p* = 0.003); *CXCR2* (2.71-fold; *p* = 0.002) and *c-myc* (1.96-fold; *p* = 0.004). Hence, we chose these four genes for downstream analyses on their potential as diagnostic biomarkers for CRC.

### 3.3. Validation of the Four-Gene Panel for CRC Diagnosis

**Figure 4:**
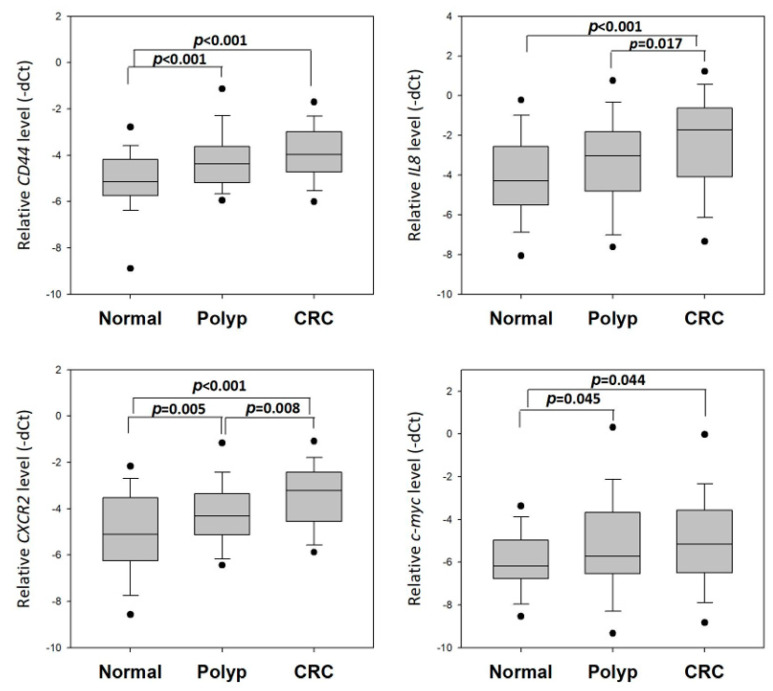
Dysregulated levels of *CD44*, *IL8*, *CXCR2* and *c-myc* among healthy controls, polyp and CRC patients in the validation study. Expression of *CD44*, *IL8*, *CXCR2* and *c-myc* in rectal swab specimens was compared among healthy controls (*n* = 60), polyp subjects (*n* = 68) and CRC patients (*n* = 45). One-way ANOVA was applied to calculate the *p*-values.

We further determined the expression levels of *CD44*, *IL8*, *CXCR2* and *c-myc* in an independent validation study consisting of rectal swab specimens from 60 normal subjects, 45 CRC subjects and 68 polyp patients. The expression of these four genes was compared among the three groups of patients (Figure 4). In line with the results in the training set, all four of these genes showed significantly higher levela in CRC patients when compared to normal subjects. The approximate fold changes of these genes in CRC patients compared to those of normal subjects were as follows: *CD44* (2.28-fold; *p* < 0.001); *IL8* (3.39-fold; *p* < 0.001); *CXCR2* (3.28-fold; *p* < 0.001) and *c-myc* (2.03-fold; *p* = 0.044). These results demonstrated that *CD44*, *IL8*, *CXCR2* and *c-myc* expressions in the rectal swabs collected from the CRC patients were indeed significantly higher than those from the non-CRC subjects.

The levels of these genes were also found to show a significant difference between normal and polyp subjects and/or polyp and CRC subjects. *CD44*, *CXCR2* and *c-myc* levels showed around a 1.30- to 1.80-fold increase in polyp subjects when compared to normal subjects, whereas the *CXCR2* and *c-myc* levels were around 0.5- to 0.7-fold lower in polyp subjects when compared to those in CRC subjects. Notably, *CXCR2* was the only gene that showed a significant difference among the three patient groups.

**Figure 5:**
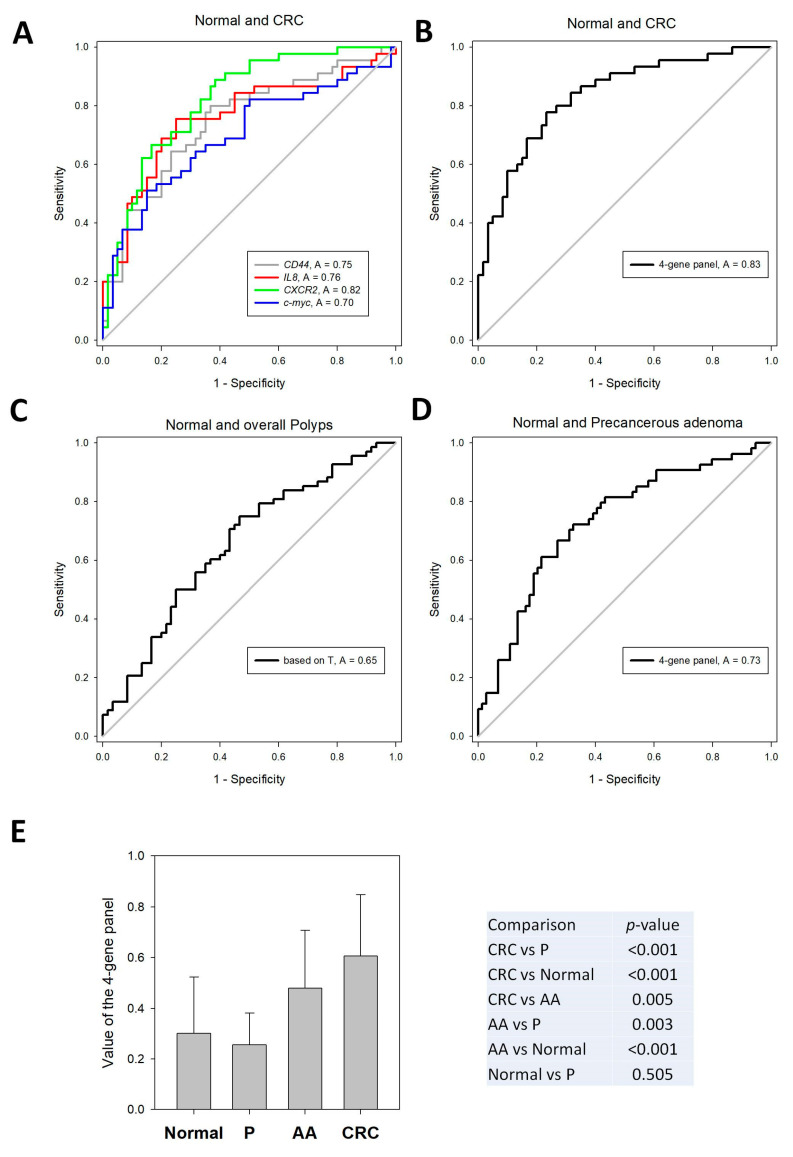
Evaluation of the performance of the dysregulated gene and the four-gene panel for the screening of CRC and polyp patients. (**A**) ROC analyses of *CD44*, *IL8*, *CXCR2* and *c-myc* for screening CRC patients from normal controls. (**B**) Multiple linear regression was applied to formulate a four-gene panel from *CD44*, *IL8*, *CXCR2* and PPARα for the screening of CRC patients. The formula was 1.033 + (0.00735 × *CD44*) + (0.0329 × *IL8*) + (0.0806 × *CXCR2*) + (0.0243 × *c-myc*). ROC analyses showed that this four-gene panel identified CRC patients from normal controls with an AUC value of 0.83. (**C**) ROC analyses showed that the four-gene panel identified overall polyp patients from normal controls with an AUC value of 0.65. (**D**) ROC analyses showed that the four-gene panel identified precancerous adenoma patients from normal controls with an AUC value of 0.73. (**E**) One-way ANOVA analysis showed that the value of the four-gene panel showed a significant stepwise increase across the normal control (N), polyp (P), precancerous adenoma (AA) and CRC subjects.

The potential of these genes as diagnostic biomarkers for CRC was analyzed by plotting ROC curves. As shown in Figure 5, all of them could significantly identify CRC patients from normal subjects (*p* < 0.001). The ROC curves had AUCs of 0.75 (95% confidence interval [CI], 0.6502 to 0.8439) for *CD44*, 0.76 (95% CI, 0.6606 to 0.8579) for *IL8*, 0.82 (95% CI, 0.7413 to 0.9002) for *CXCR2* and 0.70 (95% CI, 0.5940 to 0.8053) for *c-myc*. Furthermore, we applied multiple linear regression to formulate a combination of the four genes for CRC diagnosis: 1.033 + (0.00735 × *CD44*) + (0.0329 × *IL8*) + (0.0806 × *CXCR2*) + (0.0243 × c-*myc*). The AUC of this four-gene panel for CRC diagnosis was 0.83 (95% CI, 0.7548 to 0.9111; *p* < 0.0001). The sensitivity and specificity at the cut-off value of 0.4200 were 80.0% and 73.3%, respectively.

We next tested whether this four-gene panel could discriminate polyp patients from normal subjects. This four-gene panel was able to significantly identify polyp patients (*p* = 0.0030) with an AUC value of 0.65 (95% CI, 0.5566–0.7479). The sensitivity and specificity at the cut-off value of 0.4200 were 50.0% and 75.0%, respectively. More importantly, the performance of this panel was increased when detecting precancerous adenoma patients from normal patients (*p* < 0.001) with an AUC value of 0.73 (95% CI, 0.6433–0.8211). The sensitivity and specificity at the cut-off value of 0.4200 were 61.1% and 78.4%, respectively. Indeed, the four-gene panel showed a significant increase among the normal subjects (mean: 0.300), precancerous adenoma patients (mean: 0.479) and CRC patients (mean: 0.607) (Figure 5), indicating that this panel was able to detect the molecular changes during the transformation of normal colorectal mucosa to adenoma and, further, to tumors.

### 3.4. Predictive Value of the Four-Gene Panel

Finally, we randomly chose 58 rectal swab specimens to determine the predictive value of the four-gene panel in identifying polyp and CRC patients. Among these patients, 18 were normal subjects, 22 were polyp patients and 18 were CRC patients. The sensitivities for identifying overall polyp patients (14 out of 22), precancerous adenoma patients (10 out of 13) and CRC patients (16 out of 18) were 63.6%, 76.9% and 88.9%, respectively, whereas the specificity for normal subjects was 72.2%.

## 4. Discussion

Colorectal cancer (CRC) is a leading cause of cancer-related deaths worldwide. To improve the current situation, it is crucial to diagnose CRC patients in the early stages, as well as to identify polyp patients before their condition progresses to CRC, in order to enhance their prognosis. However, CRC is typically asymptomatic during its early stages, and symptoms often only manifest in advanced stages. Therefore, an optimal screening method is essential. Although screening programs have contributed to a reduction in mortality rates [13], none of the existing methods, including stool tests, endoscopic examinations, and imaging tests, are considered ideal due to technical limitations [14,15,16]. Recent advancements in our understanding of the molecular and cellular mechanisms of CRC have led to the development of molecular biomarkers for diagnosing CRC. Additionally, a previous study conducted in the US, which analyzed individuals with cancer, polyps or a family/self-history of cancer, found strikingly similar gene expression profiles between swab samples and samples obtained through biopsies [12,17]. In this study, we aimed to explore the potential of gene expression analysis in rectal swab specimens as a non-invasive biomarker for diagnosing CRC. Specifically, we focused on 14 genes that have been previously identified as being altered during CRC development.

We first determined whether the 14 genes selected were indeed dysregulated in CRC for our patient cohort. The expression of *CXCR2*, *SAA*, *COX1*, *PPARδ*, *PPARγ*, *Groγ*, *IL8*, *p21*, *c-myc*, *CD44* and *CSF1* was significantly higher in CRC when compared to that in adjacent normal mucosa. We also examined if the gene dysregulation in CRC can be reflected in the rectal swab specimens of the patients. The *CD44*, *IL8*, *CXCR2*, *c-myc*, PPARα, *PPARδ*, Groα, *Groγ* and *SAA* levels in tissues were significantly correlated with the levels in paired rectal swab. These results suggest that rectal swab specimens are a non-invasive means for detecting the gene dysregulation pattern in colorectal tissue.

We then performed a training study to compare the levels of these 14 genes in rectal swab specimens collected from healthy control and CRC patients. *CD44*, *IL8*, *CXCR2* and *c-myc* were consistently overexpressed in CRC patients; hence, we further investigated their levels among healthy control, polyp and CRC patients and evaluated their potential as screening biomarkers in the validation study. In line with the results in the training set, all four genes showed significantly higher levels in CRC patients when compared to normal subjects. The levels of these genes were also found to show significant differences between normal and polyp subjects and/or polyp and CRC subjects, confirming their dysregulation during the transformation of colorectal epithelium to polyp and, subsequently, to cancer.

Currently, in addition to colonoscopy, certain non-invasive colorectal cancer screening tools are available, including Fecal Occult Blood Test (FOBT), Fecal Immunochemical Test (FIT) and Multitarget stool DNA testing (MT-sDNA). The estimated sensitivities of FOBT and FIT for CRC were approximately 70%, 73.8% and 92.8%, respectively [18]. Regarding the identification of adenoma above 10 mm in size, these tests showed weaker sensitivities ranging from 23.8% to 42.4%, which were further reduced to 7.5% and 17.2% for adenoma below 10 mm [19]. Our validation and blinded study consistently showed that the sensitivities of the rectal swab four-gene panel for CRC were 80.0% and 88.9%, respectively. More importantly, the sensitivities of the four-gene panel for precancerous adenomas detection were 61.1% and 76.9%, respectively, which outperformed the current non-invasive screening methods. These results suggested that the approach of detecting molecular changes in colorectal lining through rectal swab sampling is indeed a promising biomarker for the screening of polyp and CRC. With the development of next-generation sequencing, it is expected that the sensitivity and specificity can be further improved by identifying a broader spectrum of gene expression, methylation or mutation status, particularly for pre-cancerous lesions.

Recently, more work has been carried out on discovering new biomarkers for CRC diagnosis. One source of biomarkers under extensive investigation is blood samples. One subgroup of blood-based biomarkers is the microRNA level, which was reported to have an overall sensitivity and specificity of CRC detection reaching 76%, according to a meta-analysis in 2017 [20]. Some of the best-performing panels were miR-144-3p, miR-425-5p and miR-1260b, which achieved 93.8% sensitivity and 91.3% specificity in CRC identification [21]. Yet, although the study included patients with polyps, no analysis of its performance was presented. An alternative blood-based biomarker is the methylation of genes, which epigenetically alters the expression level of targets. One commercially available test, the Epi proColon 2.0 assay, identifies the methylation status of gene SEPT9, with an average sensitivity reaching 74% among 10 studies [22]. The study also suggested that the sensitivity of such test increases only when CRC develops to a later stage, whereas a poor sensitivity of 11% was recorded for adenoma identification [23]. Our rectal swab screening method achieved not only a high CRC sensitivity but also decent sensitivities for polyp and precancerous adenoma cases. Such performance is important in removing any lesion that shows a higher potential in tumorigenesis. In addition, blood-based tests require medical staff for blood collection, and the samples must be processed within a short time (e.g., 4 h). Rectal swab samples can be preserved in RNase later buffer for a longer time, and the self-collection of rectal swab samples might be feasible, subject to further studies. Such advantage may have potential in promoting the self-testing of CRC and polyp, allowing for the earlier detection of lesions. 

Regarding the limitations of the study, one was the lack of the personal medical history (except CRC or other cancer types) of the subjects. For example, drug intakes, especially medicine that targets the gastrointestinal track, may affect the colorectal microenvironment of subjects and potentially alter the gene expression of mucosa. A family history of CRC or another cancer was also not recorded. Such information may be beneficial in understanding its impact on a patient’s rectal swab-detected gene expression. In addition, the expansion of the population for a blinded study may allow for the better verification of the sensitivity and specificity of the gene panel. Although the aim of the study was to identify any polyp or CRC using rectal swabs regardless of their molecular identities, it could be valuable if we can correlate the gene expression detected with subtypes of CRC if a larger sample size is available in a further study.

## 5. Conclusions

This study demonstrated that the use of rectal swabs sampling to detect molecular changes in colorectal lining is indeed a noninvasive screening test for the presence of polyp and CRC that can be widely applied to the higher-risk or symptomatic public through carrying out the sampling procedure in any clinics, health centers or even at home by individuals with a properly prepared kit. We believe that such rectal swab tests will be able to gain acceptance by more subjects and increase the screening rate of the public so that more CRC patients can be identified at an earlier stage and the morbidity and mortality rates of CRC can be improved.

## Figures and Tables

**Table 1:** Gender and age information for the subjects recruited in this study.

	Gender		Age	
Training study	Male	Female	≥65	<65
Normal	18	21	20	19
CRC	25	12	22	15
Validation study				
Normal	29	31	30	30
Polyp	45	23	39	29
CRC	23	22	26	19
Blinded study				
Normal	11	7	8	10
Polyp	14	8	11	11
CRC	12	6	11	7

**Table 2:** Diagnosis and stage information for the CRC patients in this study.

	Training Study	Validation Study	Blinded Study
Diagnosis			
CA Colon	27	27	11
CA Rectum	10	18	7
Stage			
I	8	11	5
II	10	10	8
III	15	21	4
IV	4	3	1

## Data Availability

The raw data supporting the conclusions of this article will be made available by the authors on request.
